# Canadian Optically-guided approach for Oral Lesions Surgical (COOLS) trial: study protocol for a randomized controlled trial

**DOI:** 10.1186/1471-2407-11-462

**Published:** 2011-10-25

**Authors:** Catherine F Poh, J Scott Durham, Penelope M Brasher, Donald W Anderson, Kenneth W Berean, Calum E MacAulay, J Jack Lee, Miriam P Rosin

**Affiliations:** 1 Department of Oral Biological and Medical Science, The University of British Columbia, Wesbrook Mall, Vancouver, Canada; 2 Department of Surgery, The University of British Columbia, West 10th Avenue, Vancouver, Canada; 3 Department of Statistics, The University of British Columbia, Agricultural Road, Vancouver, Canada; 4 Integrative Oncology, British Columbia Cancer Agency/Research Centre, West 10th Avenue, Vancouver, Canada; 5 Cancer Control Research, British Columbia Cancer Agency/Research Centre, West 10th Avenue, Vancouver, Canada; 6 Otolaryngology- Head & Neck Surgery, Vancouver General Hospital, Laurel Street, Vancouver, Canada; 7 Centre for Clinical Epidemiology and Evaluation, Vancouver General Hospital, West 10th Avenue, Vancouver, Canada; 8 Anatomical Pathology, Vancouver General Hospital, Laurel Street, Vancouver, Canada; 9 MD Anderson Cancer Center, University of Texas, Holcombe Boulevard, Texas, USA; 10 Biomedical Physiology and Kinesiology, Simon Fraser University, University Drive, Burnaby, Canada

## Abstract

**Background:**

Oral cancer is a major health problem worldwide. The 5-year survival rate ranges from 30-60%, and has remained unchanged in the past few decades. This is mainly due to late diagnosis and high recurrence of the disease. Of the patients who receive treatment, up to one third suffer from a recurrence or a second primary tumor. It is apparent that one major cause of disease recurrence is clinically unrecognized field changes which extend beyond the visible tumor boundary. We have previously developed an approach using fluorescence visualization (FV) technology to improve the recognition of the field at risk surrounding a visible oral cancer that needs to be removed and preliminary results have shown a significant reduction in recurrence rates.

**Method/Design:**

This paper describes the study design of a randomized, multi-centre, double blind, controlled surgical trial, the COOLS trial. Nine institutions across Canada will recruit a total of 400 patients with oral severe dysplasia or carcinoma *in situ *(N = 160) and invasive squamous cell carcinoma (N = 240). Patients will be stratified by participating institution and histology grade and randomized equally into FV-guided surgery (experimental arm) or white light-guided surgery (control arm). The primary endpoint is a composite of recurrence at or 1 cm within the previous surgery site with 1) the same or higher grade histology compared to the initial diagnosis (i.e., the diagnosis used for randomization); or 2) further treatment due to the presence of severe dysplasia or higher degree of change at follow-up. This is the first randomized, multi-centre trial to validate the effectiveness of the FV-guided surgery.

**Discussion:**

In this paper we described the strategies, novelty, and challenges of this unique trial involving a surgical approach guided by the FV technology. The success of the trial requires training, coordination, and quality assurance across multiple sites within Canada. The COOLS trial, an example of translational research, may result in reduced recurrence rates following surgical treatment of early-stage oral cancer with significant impacts on survival, morbidity, patients' quality of life and the cost to the health care system.

**Trial Registration:**

Clinicaltrials.gov NCT01039298

## Background

Oral cancer is a major health problem worldwide, accounting for 274,000 new cases and 145,000 deaths annually [1]. Although it occurs at a site that is easily accessible for examination it is often diagnosed at an advanced stage, with 5-year survival rates ranging from 30-60%, depending on the global locale. Treatment of early stage squamous cell carcinoma is an essential component of effective oral cancer management; a recent large (~190,000) randomized trial of a screening program showed the mortality rate ratio between intervention and control groups was 0.79 [95% CI 0.51 - 1.22] [2]. However, even with treatment there are high rates of second oral malignancies, with up to a third of these patients suffering a recurrence or a second primary [3,4]. There has been extensive research in the importance of examining the field surrounding oral cancers for risk assessment and management of this disease [5-7]. Using molecular technology, it is becoming increasingly apparent that genetically altered cells are often widespread across the mucosa of patients with oral cancer, extending into clinically and histologically normal tissue, and that these cells can drive the process of field cancerization. Recognizing this, surgeons try to remove oral squamous cell carcinomas (SCC) with a significant margin of surrounding normal-looking oral mucosa. However, the occult disease varies in size and a wealth of evidence suggests that it frequently extends beyond the tumor clearance area [5-8]. This extension may be responsible for the high rate of cancer recurrence at the primary site (10-30% of SCC cases) [9-11]. Taking margins that are too large can result in over-cutting (causing severe cosmetic and functional morbidity) and margins that are too small may leave cancerous tissue behind, as evidenced by frequent positive surgical margins and high local and regional recurrence - a failure of the 'best practice'.

Our research team has developed an approach using fluorescence visualization (FV) technology, which improves the recognition of the field at risk surrounding a visible oral cancer [12,13]. Our preliminary results show that using FV to define the field at risk for surgical resection can result in a marked reduction in recurrence rates at 3 years (0/38 vs. 7/22) [14]. Our goal in this study is to rigorously evaluate and validate the effectiveness of FV-guided surgeries compared to white light in a multicentre pan-Canadian randomized control study.

## Objective

The trial has 4 objectives. The primary objective is to determine the effectiveness of FV-guided surgery in reducing local recurrence after surgical excision of severe dysplasia or cancer of the oral cavity. Secondary objectives include 1) To collect molecular and phenotypic evidence in margins to test if FV produces a shift in surgical field, sparing normal tissue while catching high-risk occult tissue; 2) To collect relative cost-effective evidence of the two arms in both the cost per avoided recurrence and the cost per quality-adjusted life years gained; and 3) To develop a knowledge translation (KT) strategy that will foster the dissemination of FV-guided surgery across Canada and globally.

## Methods/design

### Study design

**Figure 1:**
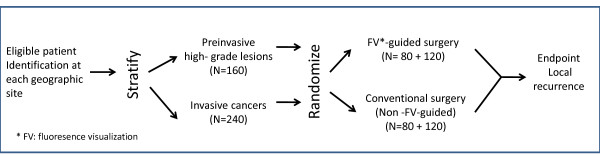
**Trial schema**. A total of 400 subjects (240 invasive squamous cell carcinoma and 160 severe dysplasia or carcinoma in situ) will be randomized equally into FV-guided surgery (experimental arm) or white light-guided surgery (control arm). The primary endpoint is a composite of recurrence at or 1 cm within the previous surgery site with 1) the same or higher grade histology compared to the initial diagnosis (i.e., the diagnosis used for randomization); or 2) further treatment due to the presence of severe dysplasia or higher degree of change at follow-up.

The COOLS trial is a multicentre, double blind, randomized controlled study, comparing FV- guided surgery (experimental arm) to conventional white light (WL)-guided surgery (control arm). See Figure 1 for the schema of the study.

This study has been approved by the human research ethics committees at each of the participating institutions. Any future amendments to the study protocol will be submitted to each committee for approval. The trial has been registered at Clinicaltrials.gov (NCT01039298).

### Sample size

A total of 400 subjects (240 invasive squamous cell carcinoma and 160 severe dysplasia or carcinoma *in situ*) will be randomized from 9 centres across Canada (from west to east): Vancouver, British Columbia; Calgary and Edmonton, Alberta; Winnipeg, Manitoba; Toronto (Sunnybrook Hospital), London, and Ottawa, Ontario; Montreal (McGill University Health Centre), Quebec; Halifax, Nova Scotia.

### Target population

The study is open to patients with high-grade preinvasive (severe dysplasia/carcinoma *in situ*) or invasive squamous cell carcinoma (T1 or T2) of the oral cavity.

### Inclusion Criteria

Patients with disease localization at oral anatomical sites that can be visualized using both white light and fluorescence visualization device (this includes ICD-10 site codes: C02.0-C06.9); patients with a clinical diagnosis of N0 or N1 as confirmed by CT scan, with the latter undergoing neck dissection; or patients with resectable locally recurrent disease diagnosed with severe dysplasia or higher grade, provided that they are at least 6 months post-treatment (this time frame will allow resolution of artefacts produced by treatment that could impact on tumor or lesion visualization).

### Exclusion Criteria

Patients with concurrent non-oral malignancy diagnosed within the past 3 years (patients with non-melanoma skin cancer or lymphoma that lie outside of the head and neck region are included); patients with evidence of distant metastasis, as determined by CT and X-ray at the time of recruitment; patients with illnesses that could preclude standard diagnostic tests and post-surgery follow-up; and patients with lesions located at the base of tongue (C01) or tonsil (C09), as these sites are not readily assessable to FV.

All patients will provide written informed consent for study participation.

### Key steps

#### Patient recruitment

**Figure 2:**
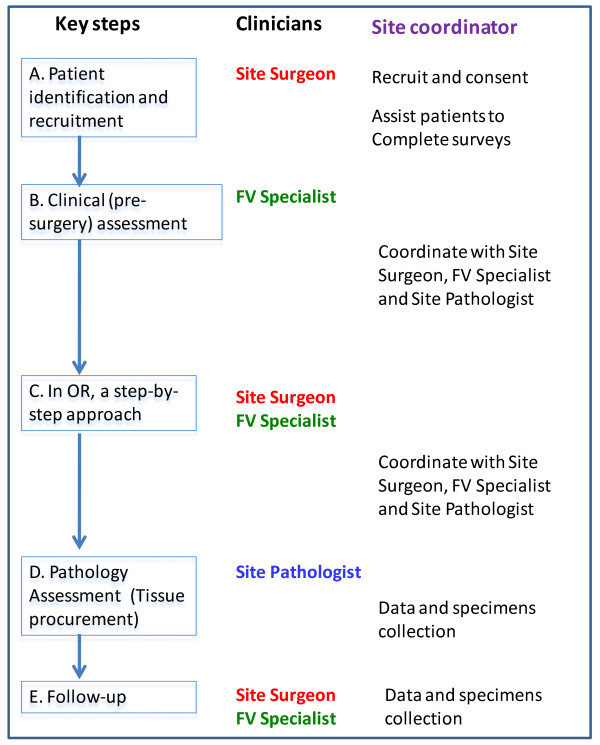
**Flow chart of key steps and the involvement of key personnel in each steps and site coordinator's role**.

Site surgeons will identify potentially eligible patients and briefly introduce the study to the patient (see key steps in Figure 2). The local site coordinator will be informed and will contact the patient to arrange an appointment to discuss the study and for a pre-surgery assessment. As part of that eligibility assessment, all patients with squamous cell carcinoma will have a CT scan from skull base to chest as a baseline to confirm the clinical nodal status and the absence of the upper alimentary and respiratory tract and lung metastasis or second primary tumor. Upon verifying all the eligibility criteria, the study coordinator will obtain informed consent from the eligible and interested patients.

#### Pre-surgery assessment

Prior to the assessment, the site coordinator will assist the patient to complete a set of study questionnaires covering socio-demographic factors, risk factors, comorbidity and family cancer history. Quality of life will be assessed with the EQ-5D [15,16], the Functional Assessment of Cancer Therapy Head and Neck Module (FACT-H&N)[17,18] and the Speech Handicap Index, a specific tool for the measurement of speech pathology. [19]

The lesion undergoing surgery will be assessed by a trained FV Specialist (FVS) using both WL and FV. The FVS will be either a dental specialist or a head & neck surgeon who will not be performing the surgery for the patient. With the assistance of the site coordinator, digital images will be obtained of the lesion under both conditions and lesion size and location will be recorded on the case report form (CRF). Both the images and the CRF will be uploaded to the trial's web-based database.

The FV assessment will be performed with an autofluorescence imaging device, marketed as the VELscope^®^, (LED Dental Inc., White Rock, British Columbia, Canada), using protocols described in Poh *et al*. [12] The examination is performed under reduced room lighting and involves the inspection of the entire oral mucosa in the same manner as the conventional intraoral examination, with special attention to the lesion site. Tissue that show a reduction in the normal pale green, appearing as dark patches, will be categorized as FV positive (FVpos). Lesions that retain the normal green autofluorescence under FV are classified as FV negative (FVneg). Both WL and FV digital images of the lesion will be recorded prior to randomization.

#### Intervention (Surgery)

At the time of surgery the operating surgeon will outline the boundary of the clinically visible lesion under white light. The FVS will take an image (***Image 1***); measure its size; and record data on the surgical tracking sheet.

##### a. If this is a FV-guided surgery (experimental arm)

With the operating room (OR) lights off, the FVS will use the VELscope Vx to examine the lesion and outline the FV change using a green Sharpie pen. After the FV positive boundary is outlined, images (***Image 2***) are taken in the dark to demonstrate the distance between FVL and clinical outline.

With the OR light back on, the FVS will measure the distance from FV boundary to the clinical boundary in 4 directions, and record this on the surgical tracking sheet. The site surgeon will outline a 10 mm surgical boundary around the clinically visible tumor and FV boundaries, whichever is wider. The FVS will take images (*Image 3*) under both FV and WL and record if there is any anatomical restriction for the placement of the standardized surgical boundary.

##### b. If this is a WL- guided surgery (control arm)

With the light off, the FVS will use the VELscope VX to examine the lesion and draw an outline on top of the surgeon's clinical boundary using a green Sharpie pen. After this step, the FVS will take another set of images (*Image 2*). In this case, the 2 outlines will be identical and this will be recorded on the surgical tracking sheet. The surgeon will outline a 10 mm surgical boundary around the clinical/FV boundary. The FVS will take images (*Image 3*) under both FV and WL and record if there is any anatomical restriction for the placement of the standardized surgical boundary.

The operating surgeon will remain outside the OR while the FVS marks the boundaries. Although it is unlikely the surgeon will remain blinded once the two markings are made, any deviation from the marked boundary will be recorded to allow assessment of deviation from the protocol.

Blood samples (5 ml in SST and 12 ml in EDTA tubes) will be collected at this time prior to the surgery. The tumor will then be resected and oriented using a suture for the anterior or right orientation. This will be indicated on the routine pathology requisition form to help the SP to orient the resected tissue. The specimen will be wrapped in a piece of cold saline gauze and kept on ice during transfer to the Pathology Department for processing.

The Site Pathologist (SP) will either use the tracking sheet or a print-out of the digital image of the excised tissue to record the tissue blocking. One-week after the diagnosis has been determined all the H&E sections will be sent to the Central Histology Review committee for histological review. If there is disagreement on the assessment between the site pathologist and the review committee, a teleconference will be arranged in order to discuss and achieve consensus. The site coordinator will upload all images, surgical and pathological CRFs and key information from pathology reports into the study database.

#### Follow-up

All patients will return for follow-up examinations every 3 months for two years and then every 6 months for the remainder of the study period. At each visit the entire oral mucosa will be examined under WL, with surgery sites photographed. The presence of lesions will be noted on lesion tracking sheets along with lesion size and location. Updated clinical information, digital images and data fields from the quality of life questionnaires will be uploaded to the database by the SC.

Decision to biopsy will depend on the clinical judgment of the surgeon, based on suspicion of a recurrence. If there is no significant clinical change, a biopsy will be taken at the surgery site at 2-year post surgery. A repeat CT scan is warranted if there is clinical suspicion of regional or distant diseases. If there is no clinical indication, Neck CT scan and a chest X-ray will be arranged at 2-year post surgery follow-up.

### Randomization

Stratified randomization will be employed. Two stratification factors will be applied: 1) institution and 2) histological grade of the primary lesion (severe dysplasia and carcinoma *in situ *or invasive squamous cell carcinoma).

A randomization program has been written specifically for this trial (JJL). According to the information collected, the central database manager under the supervision of the study biostatistician (PMB) will perform the randomization. Within each stratum, the minimization algorithm will be used to achieve balanced randomization with respect to other prognostic factors, including surgeon, gender, age, smoking history, and lesion anatomical sites. [20]

The randomization will be done 1 - 2 days prior to surgery. Only the FVS will be notified of the result. The patient, research staff, operating surgeon, and the pathologist are not aware of the assignment. The allocation list will be held by the study database manager; this individual is not involved in patient care or recruitment. The allocation will only be revealed in the event of an emergency medical situation.

## Outcome evaluation

### Primary endpoint

A composite endpoint will be used for outcome evaluation. The components are 1) local recurrence defined as a recurrence at or within 1 cm of the previous surgery site, with the same or higher grade histology compared to the initial diagnosis or 2) further treatment due to the presence of severe dysplasia or higher degree of change at follow-up. This is consistent with the current practice at all participating sites that only high-grade lesions (i.e. ≥ severe dysplasia) require further treatment. The endpoint will be evaluated and adjudicated by the Centre Pathology Review Committee (chaired by KWB) of which members are blinded to the treatment assignment.

### Secondary endpoints

In addition to the primary endpoint, we are also interested in the following endpoints.

1. Failure of the 'first pass'. A histologically-confirmed positive margin for severe dysplasia or greater histological change, either at the intraoperative or paraffin sample assessment, will count as failure of the 'first pass' margin (surgical failure). Should this happen, an additional strip of tissue will be taken during surgery or a second surgery will be performed. For the latter case, the second surgery will be done according to the patient's originally assigned surgical approach (i.e., WL- or FV-guided).

2. Regional or distant metastasis: At any follow-up time point, failure of regional or distant control, i.e., development of metastatic disease to regional lymph nodes confirmed by fine needle aspiration, CT or MRI, or subsequent pathology diagnosis.

3. Disease-specific survival: Patient's death due to disease recurrence, including failure in local, regional and distant control, is considered an event. Patients who die of causes unrelated to their oral cancer or treatment of their oral cancer are censored at the time of event. Patients lost to follow-up are considered censored at the last follow-up time.

## Statistical design and analysis

The trial data will be analysed by the biostatistician (PMB) who will be blinded to the treatment allocation (masked as Treatment A or Treatment B) during the study period.

### Sample size

Assuming the survival curves follow exponential distributions, a 25% failure rate at 3 years in the WL arm and a hazard ratio of 0.5 (i.e. ~12.5% failure rate in the FV arm), a 36-month accrual period, a minimum of 2 years of follow-up, with an overall two-sided α = 0.05, a total sample size of 350 patients is required to achieve at least 80% power using the stratified log-rank test. Assuming 10% of patients may be lost to follow-up we will randomize 400 patients.

Two interim analyses for efficacy are planned (at one-third and two-thirds of predicted outcomes) with a final analysis at the end of Year 5. A Lan-Demets spending function with O' Brien-Fleming type stopping boundaries will be employed.

## Monitoring

A Data Safety and Monitoring Board (DSMB) will meet yearly to monitor accrual and adverse events. The DSMB will also review the results of the interim analyses and provide a recommendation as to whether or not the trial should be stopped early for efficacy. The DSMB will be comprised of a medical oncologist, a head & neck surgeon, and a biostatistician.

## Fidelity of the intervention

The COOLS trial examines the efficacy of using a device. How to transfer the technique with uniformity to the participating sites across Canada is the key to success.

It will be necessary to establish uniform standard operating procedures (SOPs) for all key activities; however, each site will have its own unique environment and personnel. Prior to activation, each site will be visited with the delivery of study devices. During these visits the multidisciplinary team of surgeons, pathologists, project coordinators, and FVS at each site will be trained in all study procedures. These site visits will also provide a face-to-face opportunity to build strong relationships with site team members that should facilitate problem solving throughout the study.

Along with the site visits, a comprehensive training manual has been created and is made accessible to each site through study's web-based database. This training manual includes study protocols, operation details for each key step, how to complete and upload the scannable CRFs, sections for clinicians/surgeons with an atlas of clinical lesions in both WL and FV images and instructions for the VELscope, VELscope Vx and camera, sections for the pathologists with details in tissue blocking and the definition of key fields in the pathology synoptic report form. This manual will serve as a prototype that will be evaluated and fine-tuned throughout the project for future knowledge translation. The study website also includes a library of all study CRFs that can be downloaded.

For clinical images and the use of VELscope, all FV operators (FVS and SS) will need to pass a 2-step control process. Step 1: as part of the training process, two sets of 8 images will be provided to the site surgeons and FVS, as a first step in calibrating their judgment on clinically visible tumor boundaries and FV positive (FV loss) boundaries. The criteria for passing the step is that both clinically visible tumor boundaries and FV boundaries need to be within ± 5 mm of those drawn by the experienced FV operators from the British Columbia (BC) site. Step 2 **: **to be certified, all FV operators require hands-on experience in outlining the tumor and FV boundaries on 2 real patients. The same criteria will be used to assess this activity, with a requirement that both clinically visible tumor and FV boundaries be within ± 5 mm of those drawn by the experienced FV operators from BC site. This time, the BC specialists will draw on the images received from the study sites. This can be used to review the entire operation from new patient assessment to the completion of surgery (see Key Steps above) to ensure the high-quality operation.

After passing this 2-step control process, a site will be ready to recruit the first patient to the study. The PI (CFP) will review the first 10 patients from each site to ensure the quality of images is maintained and that mapping of clinical and fluorescence boundaries proceeds as per initial training and to provide suggestions to the site team members for any problems experienced during these activities. Subsequently, about 5% random samples from each site will be selected from time to time to check for the accuracy of boundary drawings. This can also mitigate 'margin creep' over time.

One risk is the potential contamination of endpoint by the surgeon, i.e., margin creep (expanding margins) in the control arm. We are requiring that both the FV and white light margins be justified just prior to surgery in the operating room with the presence of both surgeon and an independent party for FV assessment. In this fashion, the surgical margin will be placed in a consented and unbiased fashion. In order to keep the study double blinded and avoid potential bias, the FVS will be a different person from the SS. The periodic review of sites by central management team will also mitigate this problem.

## Data quality

The acquisition of high quality data is key to the success of any clinical trial. We have planned a systematic strategy for control of data and image capture with a special focus on ensuring that the involved processes are efficient and of high quality. For clinical data, we use a set of scannable teleforms. These forms can be scanned and quickly uploaded from the participating sites to the central database. The use of scannable forms will significantly increase the efficiency of the knowledge capture and avoid transcription errors during the data collection process. The central database manager will review uploaded CRFs for completeness and review any problems with the local site coordinators who will have access to the uploaded forms through a user interface. In order to maintain the high quality operation and integrity of the study, the CPM will periodically perform a random check of all processes of selected cases, with at least 1 case from each site every 3 months. The database will be programmed to alert the Centre and Site if a patient is overdue for their scheduled follow-up appointment. A monthly teleconference with site coordinators and site PIs will share problems among sites. We monitor the trial activity on a day-to-day basis and a common email account has been set up to alert urgent issues from the site. Any operational questions in the project will be brought forward to the Steering Committee. All problems and solutions will be logged for future reference.

A major risk to the success of this project is patient enrolment. This risk will be mitigated by requiring each site to develop patient pipeline strategies. This is a mile-stone driven project. With the consideration of allowing sufficient start-up time for each site to build its infrastructure, we project to recruit 400 patients over the first 36 months of the trial starting date.

## Discussion

The COOLS trial is a multicenter, phase III randomized controlled trial comparing 2 surgery approaches, one guided with a optical device (VELscope; experimental arm) and one without (control arm), which is different from the conventional drug trial. This is a large scale pan Canadian study to validate an FDA- and Health Canada-approved optical device to better define the surgical margin. We acknowledge the support and the shared vision from the Terry Fox Research Institute.

The uniqueness of this trial is multi-fold. Firstly, this is the first-ever pan Canadian surgical study of such calibre, involving 9 institutions across 6 provinces, more than 70 health professionals, and 400 patients. The 9 institutions are drawn from academic centres with a residency program and affiliated to provincial cancer agencies, with patient populations of interest, linkages to the dental system (referral pathway for patients), and onsite pathology services. The catchment areas of these participating sites cover over the locations from which 70% of oral cancer patients come from. Every year, there are ~3,400 patients diagnosed with oral cancer in Canada and ~60% are early stage oral cancer, which are the target population of our trial. Hence, if the results are validated, it can make enormous impact at the patient level in increasing patients' survival, morbidity, and quality of life, at the institutional level in changing the standard of care, and at the population level in improving the cure rate and reducing costs to the health system.

Secondly, this is an exceptional example of translational research from bench to bedside, from discovery to clinical implication. With the strong support of the ongoing Oral Cancer Predictive Longitudinal (OCPL) Study, BC researchers have built a strong foundation to lead this prospective, multicenter study. The OCPL study is an NIH/NIDCR-funded study which involves ~900 patients with oral precancer and oral cancer that have been monitored regularly since 1999 for progression/recurrence of disease. The OCPL study has served as a pipeline for referral of such patients to the trial in BC [21].

Thirdly, in order to change clinical practice we need to ascertain clinical efficacy through assessment of local recurrence at the clinical, histological and molecular level, assess the impact on quality of life and cost effectiveness, and construct a plan for KT. The molecular goal will examine the margin to see if the tool can produces a shift in surgical field, sparing normal tissue while catching high-risk occult tissue. The health economics goal will collect relative cost-effective evidence of the two treatments in both the cost per avoided recurrence and the cost per quality-adjusted life years gained and last but not the least, in the knowledge translation (KT) goal, we plan to develop a KT strategy that will facilitate rapid scale up of FV-guided surgery in Canada and beyond. With such a comprehensive package, the COOLS trial promises to collect evidence and information necessary for bridging the gap from discovery to clinical application of FV-guided surgery into an oral cancer solution for patients worldwide.

The COOLS trial is a large-scale study - large number of patients, types of health professional specialties, and geographically widespread institutions. The site establishment, personal training to standardize the protocol including the use of study device, and quality assurance are critical components to the success of the trial. To ensure the quality of the trial, a multidisciplinary trial coordinating centre has been developed to monitor the trial operation and a web-based database application has been created to facilitate the data, images, and sample flow. A data safety monitoring board and a steering committee have been formed to oversee the trial. Additionally, a central pathology review committee (chaired by KWB) has been formed to document and review all the margin pathology of the surgical samples and the adjudication of the endpoint based on the pathology of the follow-up biopsy.

At the time of writing, September 2011, 69 patients have been enrolled from 4 sites. It is anticipated that recruitment will be completed in August, 2013. Follow-up will be completed two years later with results available by 2015.

This trial represents a pan-Canadian partnership of sites and investigators/surgeons and is the first randomized trial of the pan-Canadian Network for Oral Cancer Control or PanCanNOC. The network, serving as the machinery for future research idea/projects, is committed to rigorously evaluating interventions in the prevention and treatment of oral cancer.

Publication and reporting date

Expect late 2015 or early 2016.

## List of abbreviations

BC: British Columbia; COOLS: Canadian Optically-guided approach for Oral Lesions Surgical; CRF: case report form; CT: computed tomography; DSMB: data safety monitoring board; FV: fluorescence visualization; FVS; FV Specialist; ICD: International Classification of Diseases; KT: knowledge translation; PanCanNOCC: pan-Canadian Network for Oral Cancer Control; RCT: randomized controlled trial; SC: site coordinator; SOP: standard operating procedure; SP: Site pathologist; SS: Site surgeon; TFRI: Terry Fox Research Institute; WL: white light.

## Competing interests

The projects received 10 units of VELscope and 20 units of VELscope Vx (cordless version of VELscope) as an in-kind donation from the LED Dental Inc., White Rock, British Columbia, Canada. The authors declare that they have no competing interests and no monetary fund received from the LED Dental Inc. or other companies.

## Authors' contributions

This project was initiated and developed by CFP, DWA. CFP, JSD, CM, JJL, MPR contributed to the design of the study and development of the protocol. DWA, PMB, and KWB contributed to the development of the trial protocol. All authors read and approved the final manuscript.

## Authors' information

**CFP**, Trial Principle Investigator, is an Oral Pathologist, a Clinician Scientist at the BC Cancer Research Centre (Departments of Integrative Oncology and Cancer Control Research) and an Associate Professor at the University of British Columbia (Faculty of Dentistry). She is the winner of the Clinician Scientist Award from the Canadian Institute of Health Research (2007-2010) and the Scholar Award of the Michael Smith Foundation for the Health Research (2007-2013). Her primary research focus involves application of molecular and imaging tools for screening, diagnosis, and management of cancerous and precancerous oral tissues. Her investigations also involve the impact of oral cancer screening in medically underserved communities. **JSD**, Trial Principle Investigator, is Clinical Professor and Acting Head, Division of Otolaryngology, Faculty of Medicine, the University of British Columbia. He is also Head and active staff of the Department of Otolaryngology, Vancouver Hospitals & Health Sciences Centre (Vancouver General Hospital and UBC Hospital), and a Consulting Surgical Oncologist, Head and Neck Tumor Group of the BC Cancer Agency. His research interests include Head and neck oncology and oromaxillofacial reconstructive surgery. **PMB**, Trial Biostatistician, is a Senior Research Scientist at UBC affiliated-Centre of Clinical Epidemiology and Evaluation (C2E2) and an associate member of the Department of Statistics at UBC. She is the Statistical Editor for the Canadian Journal of Anesthesia and has served on several grant review committees and ethics boards. Her research interests include clinical research methodology, secondary use of administrative data and statistical education. **DWA **is Clinical Professor, Division of Otolaryngology, Faculty of Medicine, the University of British Columbia. He is the Divisional Head of Otolaryngology for the Fraser Health Region Hospitals, an active staff of the Department of Otolaryngology, Vancouver Hospitals & Health Sciences Centre (Vancouver General Hospital and UBC Hospital), and a Consulting Surgical Oncologist, Head and Neck Tumor Group of the BC Cancer Agency. His research interests include Head and neck oncology and quality of life post head and neck surgery. **KWB**, Chair, Trial Pathology Committee, is an experienced head and neck pathologist. He is a Consultant Pathologist at Vancouver General Hospital. He is also a Clinical Professor with the University of British Columbia (Department of Pathology and Laboratory Medicine). He did his undergraduate training and medical school at the University of Alberta and Residency in Anatomic Pathology at the University of British Columbia. He will be the lead pathologist in the Histology Centre Review Committee and member of the Outcome Jurisdiction Committee. **CEM**, Trial Imaging Consultant, is the Head of the Integrative Oncology Department at the BC Cancer Research Centre. He is also a faculty member of both the Department of Pathology and Laboratory Medicine and the Department of Physics and Astronomy at the University of British Columbia. His research is focused on automated image analysis of cell preparations, in vivo tissue imaging, quantitative microscopy, and the development of imaging technologies for the early detection of epithelial cancer types and one of the inventors of the VELScope. He is also the co-Lead of the substudy to look for phenotypic evidence using Quantitative Pathology in margins for surgical field shift. **JJL**, Trial Design Consultant, is a Professor and Kenedy Foundation Chair in Cancer Research in the Department of Biostatistics, Division of Quantitative Sciences at the University of Texas M. D. Anderson Cancer Center in Houston, Texas. He also serves as an Adjunct Professor at both Rice University (Statistics) and the University of Texas (School of Public Health, Biostatistics). His expertise and research interests include design and analysis of clinical trials, survival analysis, longitudinal data analysis, statistical computation/graphics, statistical methods for determining drug interaction in combination studies, and cancer chemoprevention. **MPR**, Co-PI and Scientific Director of the COOLS study, is the Director of the British Columbia Oral Cancer Prevention Program (BCOCPP), a structure created within the BC Cancer Agency that has brought together ~40 clinicians and scientists of very diverse disciplines experience who are engaged in leading the evolution of system-wide change in oral cancer control in BC. MPR is a Senior Scientist in the Department of Cancer Control Research at the BC Cancer Research Centre. She also serves as a Professor at both Simon Fraser University (Biomedical Physiology and Kinesiology) and the University of British Columbia (Pathology and Laboratory Medicine). She is the winner of the Oral Health Promotion Award of the Canadian Dental Association (2010). She has extensive experience with the development and management of research projects that involve large multidisciplinary teams both globally and locally in Vancouver. She leads the NIH-funded longitudinal study, the Oral Cancer Predictive and Longitudinal study, that is developing and evaluating new technology and molecular markers for prediction of cancer progression and recurrence. She is the co-Lead of the substudy to look for molecular evidence using Loss of Heterozygosity in margins for surgical field shift.

## Pre-publication history

The pre-publication history for this paper can be accessed here:

http://www.biomedcentral.com/1471-2407/11/462/prepub
